# Supplementary material for: Identification and functional analysis of non-coding regulatory small RNA FenSr3 in Bacillus amyloliquefaciens LPB-18
Source: PeerJ. 2023 May 15;11:e15236. doi: 10.7717/peerj.15236 (PMC10194069; doi:10.7717/peerj.15236)
Supplement: Supplemental Information 4 [file peerj-11-15236-s004.zip › KO/CK-vs-T1_map/map05310.html]

KEGG PATHWAY: Asthma - Reference pathway


|  |  |
| --- | --- |
| **Asthma - Reference pathway** |  |

[
Pathway menu
| Organism menu
| Pathway entry
| Show description
| User data mapping
]

Asthma is a complex syndrome with many clinical phenotypes in both adults and children. Its major characteristics include a variable degree of airflow obstruction, bronchial hyperresponsiveness, and airway inflammation. Inhaled allergens encounter antigen presenting cells (APC) that line the airway. Upon recognition of the antigen and activation by APC, naive T cells differentiate into TH2 cells. Activated TH2 stimulate the formation of IgE by B cells. IgE molecules bind to IgE receptors located on mast cells. The crosslinking of mast-cell-bound IgE by allergens leads to the release of biologically active mediators (histamine, leukotrienes) by means of degranulation and, so, to the immediate symptoms of allergy. Mast cells also release chemotactic factors that contribute to the recruitment of inflammatory cells, particularly eosinophils, whose proliferation and differentiation from bone marrow progenitors is promoted by IL-5. The activation of eosinophils leads to release of toxic granules and oxygen free radicals that lead to tissue damage and promote the development of chronic inflammation.

|  |  |  |
| --- | --- | --- |
| Reference pathway | 184% 150% 122% 100% 82% 67% 55% | 图片下载 |
